# Community healthcare worker response to childhood disorders: Inadequacies and needs

**DOI:** 10.4102/phcfm.v11i1.1871

**Published:** 2019-04-30

**Authors:** Shanturi Naidoo, Deshini Naidoo, Pragashnie Govender

**Affiliations:** 1 School of Health Sciences, University of KwaZulu-Natal, Durban, South Africa

**Keywords:** community health care workers, childhood disability, primary health care

## Abstract

**Background:**

Community healthcare workers (CHWs) play a vital role in linking health facilities and communities where there is a high prevalence of childhood disorders. However, there is limited literature on whether this cadre of workers is adequately prepared for this task.

**Aim:**

This study explored the training needs of CHWs working in the field of childhood disorders and disabilities to improve the future training of CHWs and service delivery.

**Setting:**

This study was conducted in an urban district in KwaZulu-Natal, South Africa.

**Methods:**

This qualitative study used purposive sampling to recruit 28 CHWs and 4 key informants working in health facilities in one district of the KwaZulu-Natal Province in South Africa. Data were collected via semi-structured interviews and focus groups. Interviews were conducted in the first language (isiZulu) of the CHWs. Data were analysed thematically. Ethical clearance was obtained from a Biomedical Science Research Ethics Committee.

**Results:**

There was an evident lack of knowledge and skill in managing childhood disorders and disabilities by CHWs. Enablers and restrictors affecting service delivery were highlighted. Moreover, the training needs of CHWs have raised critical concerns because of the variable nature of training and perceived inadequate preparation for service delivery. The challenges raised were also generic to the holistic role of CHWs and not particularly specific to the CHW role in childhood disorders and disabilities.

**Conclusion:**

Training of CHWs in childhood disorders may assist in improving CHWs’ competence and confidence in the field, which may enhance service delivery and thus may assist in contributing towards improving healthcare for children at this level of care.

## Background

The South African government revitalised its focus on primary health care (PHC)^1^ in a concerted effort to manage childhood disorders and physical disabilities to achieve the goal of providing universal health care and ensuring a long and healthy life for all. Ward-based outreach teams (WBOTs), school health teams and district clinical specialist teams form pillars of the re-engineered PHC that is aimed at strengthening health promotion, disease prevention, curative, rehabilitative and early detection services.^2,3,4^ Ward-based outreach teams now include generalist community health care workers (CHWs)(for the first time) as part of the formal structure of the health service under the guidance of a facility-based nurse.^2,3,5^ Using this approach, the CHWs form the foundation of community-based PHC services, that is, extending health out of the facility through integration in assigned communities, households, educational institutions, referral networks and in providing services to persons in their homes.^2,3,5,6^ In South Africa, CHWs have been seen as important collaborators who assist the professional health care teams to deliver and strengthen services.^2,3,6,7^ For example, CHWs are expected to provide information, education and appropriate home-based care, carry out community profiling and assessments of households to identify those with difficulties or who are at risk and to facilitate referrals from the community to the clinic.^2,3,5^ Community health workers are also expected to complete follow-ups, provide psychosocial support, provide maternal and child health care, assist with problem-solving around minor health care issues and social determinants of health issues as well as to support screening as part of school health teams, Phila Mntwana centres (that is child community diagnostic centres) and to support the continuum of care of the multidisciplinary team with households assigned to them.^2,3,8,9^

In the report ‘Children with Disabilities in South Africa: A Situational Analysis 2001–2011’,^10^ the United Nations International Children’s Emergency Fund (UNICEF) acknowledged that it was difficult to ascertain the exact prevalence of children with disabilities in South Africa. In this analysis, the Stats South Africa Annual General Household Survey used the Washington Group Short Set of Questions about difficulties experienced in seven domains of functioning in children, namely vision, hearing, mobility, memory, concentration, self-care and communication.^10^ According to UNICEF, it was estimated that there are 474 000 children living with severe disabilities in South Africa, with more widespread mild to moderate disability amongst children.^10^ According to the KwaZulu-Natal strategic plan (2015–2019), the most common reasons for admission in children included gastroenteritis (21%), pneumonia (20%) and neonatal conditions (14%) including sepsis, jaundice, preterm delivery or low birth weight, respiratory distress syndrome and congenital pneumonia.^11^ Moreover, the Child Health Problem Identification Programme (2010) indicated infant and under-five mortality rates in Kwao to be 32.9/1 000 and 44.6/1 000 births, respectively.^11^ HIV and severe malnutrition accounted for a large percentage of infant mortality during this period (51.4% diagnosed with HIV and 31% diagnosed with severe acute malnutrition).^11^ From this data, it appears that 1 in 22 children born in KwaZulu-Natal Province die prior to their fifth birthday, which indicates the need for integrated community-based programmes.

The National Health Insurance,^12^ introduced as a funding system to support universal health coverage, National Service Delivery Agreement^13^ and the National Development Plan^14^ emphasise the need for a re-engineered PHC approach and the need to reduce maternal, infant and child mortality through the provision of care to families and communities. The role of CHWs, currently employed by both the Department of Social Development and Department of Health (DoH) has evolved from a volunteer position to a key team member in delivering the re-engineered PHC services in the health sector.^7^ Refer to Table 1 for a synopsis of some of the key policies. In 2003, CHWs were employed via the Expanded Public Works programme.^7^ By 2006, the DoH attempted to standardise and accredit CHW training through registration of CHW qualifications in terms of the National Qualifications Framework (NQF).^15^ The Policy Framework and Strategy for WBOT (2018) has finally formally integrated CHW into the health sector^3^ with the agreement on the standardisation of remuneration for CHWs in the DoH being formalised by the public health and social development sectoral bargaining council in June 2018.^16^ This agreement argues for the need to develop standard operational procedures for recruitment, placement and skills development for CHWs.^16^ These echo sentiments articulated in the Policy Framework and Strategy for WBOT.^3^

**TABLE 1:** Policies relevant to this study.

Description and goals	Application to this study
**National Health Insurance**^**12**^	
*Policies* on the National Health Insurance (NHI) are envisioned to address healthc are challenges experienced by children within South Africa. The NHI is a financing system that proposes a system that would allow for affordable access to health care for all South Africans irrespective of socio-economic status. Two of these priority programmes are the CHW programme and the rehabilitation programme.	Polices have shown that the government has realised the importance of rehabilitation and CHW services and the interrelated nature of the two services since 1994. There is no current assessment of rehabilitation programmes or of CHW programmes in KZN, which makes it difficult to assess the programmes in light of the aims and objectives for each as set out by the NHI.
**National Service Delivery Agreement**^**13**^	
*The National* Service Delivery Agreement (NSDA) is a charter that reflects the commitment of key sectoral and intersectoral partners related to the delivery of identified outputs as they relate to a particular sector of government. The government agreed on 12 key outcomes, which will be utilised as the key indicators to guide the programme, which was said to be the action plan for 2010 to 2014. The priority of the NSDA was to improve the population’s health and abide by the government longevity and good health for all South Africans.	There were four main outputs, which included increasing life expectancy, decreasing maternal and child mortality, combating HIV and AIDS and decreasing the burden of disease from tuberculosis, and strengthening health system effectiveness.
**National Development Plan**^**16**^	
*The government’s vision* for the development of health and welfare in SA is encompassed in the NDP 2030. This document includes two goals that address childhood disorders, namely: Goal 3: Reduced maternal, infant and child mortality. Goal 7: Primary Health Care Teams provide care to families and communities.	This shows that there is a clear need for CHWs in order to provide care to families and communities. This illustrates that CHWs are needed to reduce the maternal, infant and child mortality rate.
**KwaZulu-Natal Strategic Plan ^11^**	
*The vision of the* strategic plan is ‘optimal health for all persons in KwaZulu-Natal’. The mission is to develop and implement a sustainable, coordinated, integrated and comprehensive health system at all levels, based on the primary health care approach through the District Health System.	The strategic plan emphasises maternal and child well-being and the reduction of child mortality, which is aligned to the Sustainable Development Goals. However, this does not speak of intervention required when these children survive or have consequences such as cognitive impairments or developmental delay because of severe malnutrition.

CHW, community health worker; KZN, KwaZulu-Natal; SA, South Africa; NDP, National Development Plan.

Despite the progress achieved with the integration of the CHWs into the formal structure of the ward-based team, there remains ambiguity over the role and scope of CHWs’ tasks as the current ward-based teams are generally top–down, vertical and disease-orientated programmes with challenges arising from poor supervision, inadequate and fragmented disease-specific training.^17,18^ Moreover, different interpretation and implementation of the CHW scope, allocated tasks and working conditions are evident in different provinces.^17,18^ The National DoH (2015) authorised a rapid assessment of WBOT in seven provinces to understand the challenges and to generate potential solutions.^18^ The rapid assessments found that WBOT programmes contribute towards maternal and child health, adherence support and home-based care.^17^ Despite the rapid assessment of WBOTs finding support in the facility-based extension model, some authors suggest that the current system creates tension in the PHC system, as nurses have to juggle their clinical-based responsibilities with the supervision of CHW, which leads to gaps in CHW supervision.^17,18^ Furthermore, there has been insufficient attention focused on infrastructure, provision of materials, leadership and accountability.^17,18^ Community health workers are also allocated to school health teams and Phila Mntwana centres, where the aims are to ensure the early identification of children with malnutrition, tuberculosis, disabilities, early childhood development and other health conditions as early as possible.^8,9^ Marcus et al.^18^ suggest that a community-orientated primary care model may be a better option to adopt. Community-orientated primary care teams would consist of locally available health care professionals involving a nurse, social worker and other cadres of health workers such as occupational, physical and audio and speech therapists where necessary, in a geographically defined area, responding to the needs of the community rather than the PHC facility’s priorities. Though this may be financially more expensive, it would ensure that the CHWs’ health care capability is developed and supported though ongoing work-integrated learning.^18^ This appears to be supported in principle by the *Policy Framework and Strategy for Ward-based Primary Healthcare Outreach Teams 2018/2019*.^3^ This policy states that ‘six to ten CHWs must be part of the multidisciplinary team and their health promotion, disease prevention, therapeutic, rehabilitative and palliative care must be supported by a health practitioner in PHC and environmental officers in the community’.^3, p. 15^ The implementation thereof remains to be seen. Currently there also remains limited literature in terms of how CHWs respond to childhood disorders and their perceptions of their confidence in addressing these conditions.

### Current role of community health workers in childhood disorders and training

**FIGURE 1:**
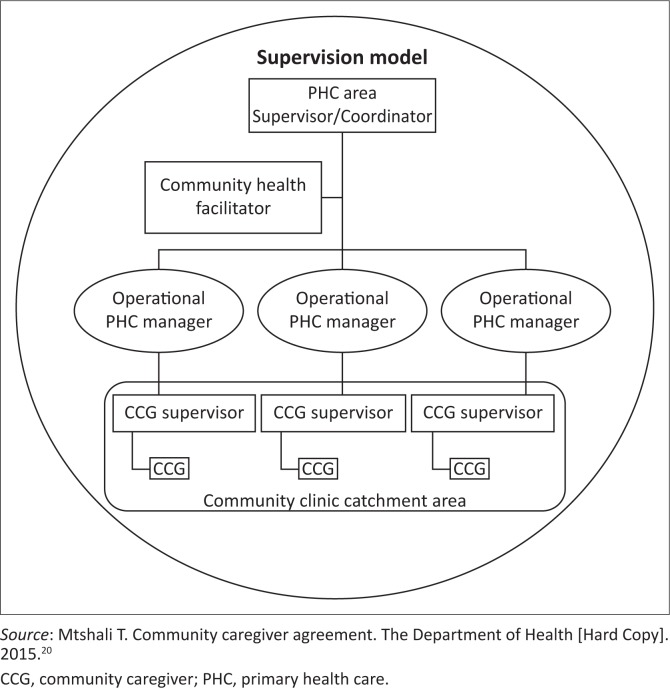
Organogram – supervision model.

The National DoH contract for CHWs, formulated in 2015,^5^ highlights expectations of CHWs in their respective communities, with regard to children specifically. Several authors have reiterated that CHWs contribute towards maternal and child health (Figure 1).^8,19,20,21,22^ However, a common theme that is evident in the literature remains that CHWs are not adequately trained for their tasks, with variability in the training noted.^3,18,23,24^ The Community Care Worker Policy Management Framework^5^ proposes that ‘there are applied skills programmes rather than qualifications (for community care workers). Therefore the skills programmes can be defined as job specific and workplace-specific’ (Table 2).^5, p. 69^

**TABLE 2:** Contract requirements and training areas for community health workers.

Contract requirements of CHWs in childhood disorders^5^	Training areas based on Community Care Worker Policy Management Framework^5, p. 69–71^
Carry out delegated health activities including but not limited to areas such as malaria, tuberculosis (TB) treatment and control, HIV and AIDS, rehabilitation, hypertension and diabetes management and control, in both adults and children Identify acute and chronic illness and facilitate relevant referral, treatment and care, in both adults and children Distribute health information to community members (health promotion material and information), including but not limited to delivering comprehensive packages of community maternal, neonatal, child, women’s health and nutrition Conduct household assessments to determine local health needs and plan interventions as well as encouraging communities to take increased responsibility for their or their child’s health Link community members (including children) to resources Identify orphans and vulnerable children, refer them and do follow-ups accordingly	Communication skills in active listening, verbal and non-verbal communication and techniques to provide basic health and social development information Sound understanding of human and health rights and ethical practice, a general understanding of the health and social development systems Orientation to primary health care and social development services and structures Ability to identify, map and utilise community referral networks Basic life support (first aid) Have an understanding of prevention, screening, management and treatment of HIV and AIDS, TB and chronic illnesses, including support of chronic care programmes and identifying danger signs of key chronic conditions Support people with disabilities with daily living activities and access to services Support older persons with daily living activities and access to services The identification and emergency management of childhood illnesses as part of integrated management of childhood illnesses Basic nutrition and food security Identify, support and care for orphans and vulnerable children Support people with disabilities with daily living activities and access to services

*Source*: National Department of Health. Community care worker management policy framework, Version 6.0 [homepage on the Internet]. 2009 [cited 2018 Dec 01]. Available from: http://www.cabsa.org.za/sites/default/files/2009%20-%20Community%20Care%20Worker%20Management%20Policy%20Framework.pdf.^5^

CHW, community health worker.

The 10-day training through the KZN DoH covers the following modules: *Being a Community Health Worker, Situational Analysis, Environmental Health Care, The District Health System, Primary Health Care Concept, Acute and Chronic Diseases and Their Management at Home, TB and Directly Observed Treatment, Short Course Body Systems, HIV and AIDS Including Counselling and Support, Health Education and Promotion of Maternal and Child Health, Integrated Management of Childhood Illnesses, Prevention of Mother-to-Child Transmission, Anti-Retroviral Treatment, Disabilities, Infectious Diseases, Community Development*.^20^ Despite the policy being in place, the implementation of the training is variable, including the mentoring and supervision offered to classify this as workplace-specific training. Moreover, based on the expectations highlighted in Table 2, a disjunct between the contract requirements, training areas and actual training is clear.

Given the acknowledged role of CHWs in childhood disorders and the prevalence of childhood disorders and disabilities in the eThekwini District,^11^ there was thus a need to explore whether CHWs, who are considered the ‘backbone of the primary health care system’, are adequately trained to deal with this specific group. This study was thus aimed at determining the training needs of CHWs in respect of childhood disorders and disabilities in eThekwini within the PHC system and National CHW Policy Framework in light of the shifting of PHC functions, from professional health care workers to CHWs. This article serves to identify gaps and help improve future training of CHWs and the service delivery offered by CHWs for childhood disorders and disabilities.

## Methods

### Study design

This study followed a descriptive qualitative design^25^ to determine the training needs of CHWs in childhood disorders and disabilities. A qualitative design was appropriate for this study, as multiple perspectives were required to understand the training needs of the CHWs through in-depth information from focus groups with CHWs and key informant interviews.^17^

### Setting

The study was located within three purposively selected peri-urban communities (Lamontville, Cato Manor and KwaDabeka) within the eThekwini Municipality of Durban, in the KwaZulu-Natal Province of South Africa.

### Study population and sampling strategy

Two stakeholder groups, namely CHWs (*n* = 28), CHW managers including supervisors (*n* = 3) and a DoH management representative (*n* = 1) were included in the study via purposive sampling of individuals who met the selection criteria. Participants were informed of the study by the first author at their respective sites and invited to volunteer to participate. Specific criteria for selection of the CHWs included those volunteers who (1) were employed by the KZN DoH, (2) executed their work within the eThekwini District and (3) were employed for a minimum of 1 year. The main criterion for the key informants necessitated that the individual be employed by DoH and involved in either the supervision or training of CHWs.

### Data collection

**FIGURE 2:**
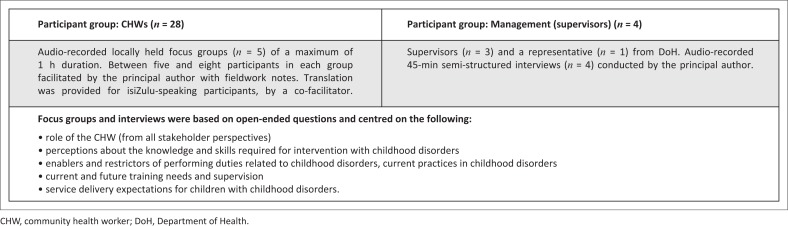
Sample and data collection methods.

Two methods of data collection were utilised in this study, namely focus groups and semi-structured interviews (see Figure 2). The timing of the data collection was concurrent, with the data merged for interpretation. Informed consent was obtained from each individual prior to commencement of the focus group and semi-structured interviews together with demographic information. The focus group was piloted with five CHWs, which allowed the schedule of questions used to be revised to ensure that the questions were easy to understand and to prevent ambiguity.

### Data analysis

All audio recordings were transcribed and checked for accuracy against the recordings by the principal author. A total of nine transcripts were available for analysis. The data were read and reread to identify the first level of coding using thematic analysis.^25,26^ Each transcript was analysed separately with initial descriptive coding. Reduction into categories followed with memos for the themes and subthemes.^25,26,27^ Verbatim quotes were utilised to maintain the voices of the participants and provide thick descriptions. Upon reaching the fifth focus group it was determined that a level of redundancy had been reached, as similar issues were repeatedly raised in the focus group discussions, and sampling ceased.

### Trustworthiness of the study

Trustworthiness was ensured by adhering to the principles of credibility, dependability and confirmability. Participants with various experiences were selected for the study through purposive selection, which increased the value of the data collected. Credibility was confirmed by ensuring the views of the participants were accurately captured using audio-recording devices. Dependability was ensured through keeping a record of the processes of data collection and methods of analysis. To ensure confirmability (that the view of the participant was represented rather than the view of the researcher), bracketing was completed by the principal author data collection and analysis process.^28^ Bracketing refers to the process of being unbiased and accurately reporting the experiences and data during the research process.^29^

### Ethical considerations

Ethical approval was obtained from the Biomedical Research Ethics committee (BE223/17). Gatekeeper permission was sought from the district and provincial offices of the Department of Health as well as the facilities (hospitals and clinics) to which CHWs were assigned. Consent was obtained for audio-recording and for participation, as it was voluntary. Principles of anonymity and confidentiality as well as justice were maintained throughout the study.

## Findings

The participants in this study included CHWs (*n* = 28), and key informants (*n* = 4).

### Community health workers

All participants were female (100%) with a majority of the participants (50%) between 40 and 49 years. Only two participants had been upskilled, with NQF levels of 1 and 3. A majority of the participants (43%) had a maximum of a Grade 11 education. A large percentage (64%) of the CHWs had 6 years of work experience in their role as CHW. All participants (100%) have exposure to childhood disorders in their daily practice; however, only a quarter (25%) had received training in the area of childhood disorders.

### Key informants

Three of the four key informants had completed postgraduate training. Experience ranged between 2 years and 7 years. All participants had been exposed to childhood disorders as part of their job functions, of which only two had received training. Three of the key informants were supervisors of the CHWs with the remaining informant occupying a senior management position of ward counsellor.

**FIGURE 3:**
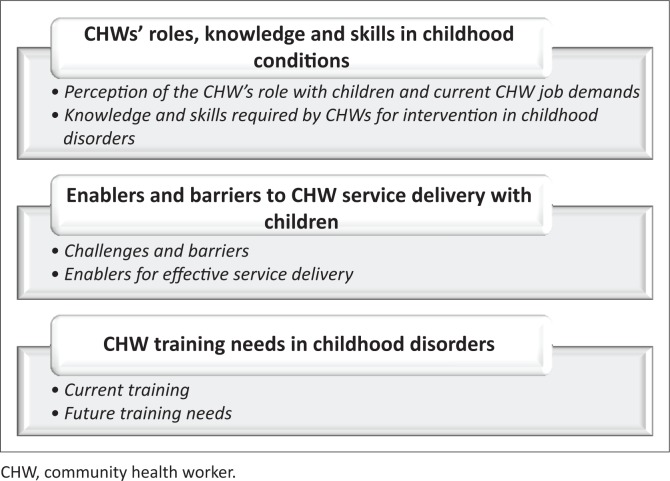
Summarised themes and subthemes.

Four main themes, each with respective subthemes, emerged. These are summarised in Figure 3. The views of all participant groups are reported under each theme. The participants used the term *CCG* often to represent *CHW*, hence the use of the acronym CCG (community caregiver) in the quotes.

### Theme 1: Community health workers’ roles, knowledge and skills in childhood conditions

The CHWs reported their role in children’s conditions and their perception of the knowledge and skills required to deliver a service. Each of the subthemes is outlined in the following content, with relevant quotes.

#### Community health workers’ perceptions of their role with children and their current job demands

Community health workers perceived that their role in childhood disorders and disabilities stemmed from the *Phila Mtwana* programme. This is a programme meaning ‘healthy baby’ that was established to enhance breastfeeding, child immunisation and child monitoring. The Phila Mtwana project was reported to be effective in identifying children with problems and referring them for help to the local hospitals or clinics. Identification of children with growth or weight problems (Severe Acute Malnutrition & Moderate Acute Malnutrition), using the MUAC l (mid-upper arm circumference) tool, and the resultant referral was viewed as another role:

‘It’s where we go to sites for Phila Mtwana; they take the children’s weight and they also give children supplements.’ (Participant 1, female, CHW focus group 5)

‘If we find a child under 5, we measure his/her growth using MUAC and see if the child is growing well. If the colour is red, that means the child is in danger; if it’s yellow, that child is not growing well, and if it’s green, we see that the child is growing well.’ (Participant 3, female, CHW focus group 4)

Community health workers reported that when they entered a home with a child with a disorder or disability, they obtained information about the child including biographical information, the medical history including any diagnosis obtained, surgeries and treatment received, and current intervention, which may include rehabilitation, medication and future appointments, amidst others. They educated the mother or caregiver on basic information on care for the child, activities of daily living (bathing, dressing, grooming, feeding and administration of medication) and how to effectively physically handle the child. Additionally, CHWs identified potentially vulnerable children, who were then referred to the respective stakeholders for intervention. These referrals were dependant on the needs of the children, which varied from school placement to retrieval of birth certificates and application for grants:

‘So, we go the home and we ask about the child, if they are going to the clinic, what kind of medication they are on, what kind of treatment that the child is on, and if there is nothing that the child is getting then we refer to the clinic.’ (Participant 1, female, CHW focus group 4)

‘… and teach the mother how to handle the child. And not to scream you know … we just educate … just to understand every reaction.’ (Participant 2, female, CHW focus group 3)

‘… we search for the vulnerable children, and know that they have birth certificates. And they are getting grants.’ (Participant 2, female, CHW focus group 2)

Community health worker supervisors reported that CHWs were expected to take a medical history and that the supervisors advised the CHWs to refer children who were not accessing interventions to a clinic. Additionally, the CHWs played a role in identifying assistive device needs for children, which involved locating children requiring wheelchairs, referring them to wheelchair organisations and thereafter assisting in the distributing of wheelchairs to the respective children. If they were unable to obtain a wheelchair from an organisation, a second-hand wheelchair was located in the community. Furthermore, CHWs were expected to assist with the collection of medication for children who weren’t able to access the clinic or hospitals and educate the caregivers on adherence to these requirements:

‘Maybe if it is a cerebral palsy child, obviously they have epilepsy; if there is treatment, then they come with all that history.’ (CHW facilitator, female, key informant interview 3)

‘… it is to teach the family how to manage the child [disabled child] and then whenever they need to refer like to the clinic and then they write a referral letter.’ (CHW facilitator, female, key informant interview 3)

‘He was a 15 years old boy that has a cerebral palsy. He has a need of a wheelchair. We also work in relationship with stakeholder. There was an organisation called APD [Association for the Physically Disabled]; they assist us with wheelchairs. So it was a successful programme with them.’ (CHW facilitator, male, key informant interview 1)

‘… to teach them how to take their medication, adhere to their medication for their children.’ (CHW supervisor, female, key informant interview 2)

#### Knowledge and skills required by community health workers for intervention in childhood disorders

All of the CHWs expressed a concern about their lack of knowledge and skill with childhood disorders. This lack of knowledge and skill resulted in the community members becoming despondent about the CHWs’ ability to help their children and the concomitant demotivation noted in the CHWs because of their inadequate training and competence. Community health workers reported that there was no consistency in the training provided and there were different topics covered in the different training courses offered:

*‘Ulwazi*, knowledge, knowledge *asinayo* [knowledge, we don’t have knowledge].’ (Participant 1, female, CHW focus group 4)

‘I am feeling bad because I don’t know how to help the child.’ (Participant 3, female, CHW focus group 2)

This perceived lack of knowledge and skill is further depicted by the following scenario:

‘Yeah … on my neighbour there’s a child with the fits … Epilepsy … I didn’t know everything about the person with the epilepsy … what to do … exactly so if we can just get enough training or knowledge about this thing … Cause others they say … put the key in the mouth … I do … cause I didn’t know … so they call me … there’s a child with the fits … put the key in the mouth … I put the key in the mouth.’ (Participant 2, female, CHW focus group 1)

The CHW supervisors found that educational levels played a role in the CHWs level of performance and attainment of knowledge and skill. Furthermore, CHWs received training at different times and there was a lack of consistency in the training. The timing of the training resulted in some knowledge being deemed to be out of date, for example, the training completed with older CHWs. The CHW supervisors reported that not all of the CHWs had received training on childhood disorders and that the younger CHWs utilised technology to research information, but this was not true for the more senior CHWs:

‘Others, they will see this as a problem but not sure what area is the problem. But she can see that child has got a problem, but she wouldn’t know that this is a Down syndrome. But the child is not normal from the other children. So that’s why I’m saying not all of them have the knowledge.’ (CHW facilitator, female, key informant, interview 3)

The DoH representative confirmed that there was minimal knowledge and skill in childhood disorders and disabilities amongst CHWs:

‘… they really need training on children because they do come across such things and they don’t know how to manage them.’ (DoH representative, key informant, interview 4)

### Theme 2: Enablers and barriers to community health workers’ service delivery with children

The CHWs and their supervisors reported that there were several factors that restricted and enabled their ability to deliver services to children.

#### Challenges and barriers

The CHWs and their supervisors reported that they were challenged with a lack of staff, lack of transport, being unclear about the role of other stakeholders and lack of supervision. Another issue raised was the lack of cooperation from relevant stakeholders because of the CHWs’ role not being recognised. Other stakeholders in this case refers to health practitioners such as rehabilitation members, doctors and nurses. Moreover, the community members often do not provide the CHWs with access to their homes and turn them away. The CHWs and supervisors attribute the community members’ refusal of assistance to the stigma still attached to disability because of cultural beliefs:

‘… sometimes the mother doesn`t open the home for you as a CCG due to personal issues, and sometimes even if you go to the home, rocks are thrown at you.’ (Participant 4, female, CHW focus group,group 5)

‘Some of the patient, they are so shy. Some of them, they have to lock their child in the room … there’s a lot of issues they’ve got.’ (CHW facilitator, male, Key informant, interview 1)

‘… maybe the child is disabled, then they don’t want other people to see those children. The family stop the CCG.’ (DoH representative, Key informant, interview 4)

The CHWs reported that supervision was generally insufficient. Additionally, some CHWs reported that their supervisors lacked the knowledge and skill to provide adequate assistance when needed. This was attributed to CHW supervisors in some sites having the same training as other CHWs but some having more experience:

‘We use to ask the supervisor … I think they [are] just like us … they [are] blank like us.’ (Participant 2, female, CHW focus group, group 1)

According to the DoH representative, supervision is insufficient as there are not enough CHW supervisors. The training received by CHWs and CHW supervisors is the same. There is seldom further training offered for CHW supervisors:

‘… the training is the same because we do not separate them unless there’s a training for supervisors.’ (DoH representative, key informant, interview 4)

#### Enablers for service delivery

The CHWs reported that they found their supervision sessions to be beneficial. Supervision is provided by CHW supervisors, which may include outreach nurses, the matron in charge of the clinic or CEO of the respective clinic or hospital. Support is provided from the supervisors through WhatsApp groups and home visits.

‘Whenever they have a problem they come and report to us, and then we go and then we see what we can do.’ (CHW facilitator, female, key informant, interview 3)

According to the DoH representative, there is an organogram that is formulated for CHWs:

‘… it is the CCG, the supervisors, the community health facilitator, the clinic manager (the operations manager), we have PHC manager, are the ones involved in that.’ (DoH representative, key informant interview 4)

Community health workers reported that they had a passion for their job, an internal motivation that kept them going. The majority of CHWs stated that they loved what they do and wished to help their communities. Their motivation for their job grew when they were able to see progress in their clients:

‘[It] is because we [are] passionate to do the work [that] we always help, and if there’s no one who will take care of the person, we just do the job. We don’t say that we are teaching or what; we just do the job.’ (Participant4, female, CHW focus group 2)

‘Anything else that makes me keep going is cause I love my jobs … I didn’t go there because I’m aiming [for] money … I went there because I want to help my community.’ (Participant 4, female, CHW focus group 1)

Community health worker supervisors concurred that CHWs displayed a passion for their job:

‘I can say it’s the passion because it comes from your heart.’ (CHW supervisor, female, key informant interview 2)

### Theme 3: Current and future training needs

The CHWs and their supervisors outlined the current training that is received and their views on the future training needs required to enable improved service delivery to children and their caregivers.

#### Current training

Most CHWs reported receiving 2-weeks (10-days) home-based training that was held throughout eThekwini in 2015. This training was based on the community needs and did not necessarily cover health-specific issues. Thereafter there was no other training offered from the DoH. All of the training attained varied according to the area the CHW was placed in; therefore the content of the training sessions varied:

‘If we can get more trainings, not 10 days training.’ (Participant 2, female, CHW focus group 1)

‘… we are not doing the same training.’ (Participant 3, female, CHW focus group 3)

Supervisors stated that there were clear gaps noted during meetings and case management.

‘… usually in the second month we are having meetings whereby we are collecting the information … so thatʼs where we feel that we see the gaps.’ (Male, key informant, interview 3)

According to the DoH representative, training was received from MATCH (Maternal, Adolescent and Child Health) but this was community-based and not health-specific. Childhood disorders were not covered. Community health workers reported that they used their past experience and exposure to childhood disabilities to respond to the needs of the children. The DoH representative noted that the lack of funding hindered the DoH’s ability to host further training.

‘… they are specific on the community … anything based on the needs of the people … the training from MATCH, it covers everything but they are not specific to health …’ (DoH representative, key informant, interview 4)

‘… even if you have a wish list to do the training, if you don’t have the money …’ (DoH representative, key informant, interview 4)

#### Future training needs

The CHWs and the key informants concurred that there is a critical need for training in identifying and dealing with childhood disorders and disabilities. Community health workers stated that they required basic information on different childhood disorders so that they may be able to better identify these children and make appropriate referrals to the relevant stakeholders. Handling of these children is also noted as critical, as CHWs reported being ill-equipped in their handling skills of children with disabilities. In addition, they requested training in first aid and health and safety, as they viewed this as a basic skill required in assisting vulnerable children. The CHWs and key informants indicated that future training plans should include sign language, physiotherapy exercises, epilepsy, mental health, the role of the different rehabilitation members and how to assist a blind person. Community health workers reported a lack of knowledge with regards to community resources, for example, special schools and organisations available to better assist children in their respective areas. Furthermore, the CHWs stated that they required assistance in dealing with how to enter a home where the caregiver does not want to receive their services.

‘… because that means the child has a different disease and I don’t know how to handle it or deal with the child so I must be trained first.’ (Participant 1, female, CHW focus group 5)

‘… we don’t know first aid but we do it …’ (All participants, female, CHW focus group 1)

‘I think we have to know where exactly we must refer a person … or who we must call …’ (Participant 2, female, CHW focus group 1)

According to the DoH representative, plans are in place to initiate training of CHWs using an external service provider:

‘… but soon Zoe life, we have agreed that they are going to train them because they are specially targeting those children at the community.’ (DoH representative, key informant, interview 4)

## Discussion

Firstly, in terms of the CHWs’ roles, knowledge and skills, both the CHWs and their supervisors identified that the CHWs were expected to participate in the Phila Mntwana programme, that is, to identify children with difficulties either at risk (growth and weight) or who have a disability. There was agreement amongst participants that CHWs were required to act as health educators, which concurs with available literature.^8,9,23^ Moreover, as mentioned in the WBOT policy strategic plan^3^ the CHWs were required to facilitate referrals and linkages to clinics. However, other participants felt that they were required to facilitate health service delivery and to provide doorstep services, including the collection and delivery of medication for children, although these activities were not part of their official CHW contract. Furthermore, there was an expectation that CHWs needed to identify and provide assistive devices such as wheelchairs and refer children to the relevant health care professional. This indicates that there is some confusion regarding CHWs’ roles and responsibilities and highlights that the current training does not adequately prepare the CHWs for the expected service delivery. Austin-Evelyn et al.^30^ reported that CHWs face challenges of inconsistent expectations, as well as a vague or inappropriate scope of work. It is clear that the role confusion and the lack of knowledge and skills had a direct effect on the CHWs’ value within the health care system, which is in keeping with the available literature.^31,32,33^ One of the strengths of the CHWs’ role is the rapport that is developed with community members, which facilitates a better understanding of the community members in terms of their social and behavioural experiences, and which can potentially aid or hinder the intervention process and may assist with strengthening service delivery.^32^ However, the CHWs require clear guidelines or standard operating procedures in terms of their role and scope when responding to children with childhood disorders and disabilities to ensure that training can be tailored to these needs and to ensure that CHWs are fit for purpose.

Secondly, CHWs are often the overlooked member of the health care work force.^32,34^ Because of the challenges experienced by CHWs both from health care workers and caregivers, CHWs tend to question their relevance. Community health workers in this study reported that they received no cooperation from health facility staff, as they view them as insignificant, which is possibly because of the non-integration of CHWs into the health system and confusion about their role. This finding concurs with the available literature, as CHWs are perceived as lowly aides and not simply integrated as fellow staff members with other clinic staff.^6,7,32^ Additionally nursing personnel are often unwilling to aid caregivers of children who are referred by CHWs; therefore, children with disorders and disabilities are often not given access to interventions that are available. All cadres of health care professionals need to receive training on the role of CHWs and vice versa so that relevant referrals occur in order to prevent secondary complications. According to Jenkins et al.,^34^ effective and regular supervision could help meet the challenges specifically to CHWs, especially in their conversion from volunteers to CHWs.

As much as the organogram reflects the reporting pathway of a CHW, this is not always adhered to, as the following tier of supervision, which is normally a nurse, is often of the view that the supervision of CHWs is either not their functional responsibility or is a low priority function.

The DoH makes provision for the supervision and accountability of CHWs through the deployment of a CHW supervisor, the health facilitator, the clinic manager and thereafter the PHC manager. Community health workers in this study reported that this supervisory mode, when put into practice, is beneficial. Community health worker supervisors voiced that attempts to better communicate with CHWs on the ground level can assist CHWs in dealing with children. Other supports suggested included a monthly debriefing of the CHWs and more regular peer or supervisory support to assist CHWs with applying their knowledge of service delivery and the effect that the services they deliver generates. Despite the many challenges experienced by CHWs, their commitment and passion in assisting vulnerable children drives them to continue striving to achieve desired results.

Thirdly, current and future training needs for CHWs were noted. According to the DoH representative, all CHWs had been trained on the reported curriculum, which was completed over a 10-day duration. This training was based on the community needs and did not necessarily cover all of the topics listed, nor did the topics have a health-specific focus. No further training was provided by the DoH because of financial constraints. Supervisors stated that there was a great need for training for all CHWs in eThekwini as there were clear gaps noted during meetings and case management discussions. The training attained varied according to the prevalence in each area and available resources. Some CHWs received training from nurses within their institutes and some from organisations that were willing to provide training. These trainings varied in length, some being a few hours and others a few days.

The challenges currently experienced by the CHWs in this study when dealing with children reflects the inadequacy of their current training programme. Despite CHWs having a clear perception of what was required of them when they approached households with children with disabilities, they encountered challenges for which they had not been trained, which resulted in the community losing faith in their capabilities. This has been documented in a previous study with CHWs in one of these communities.^33^ The findings of this study concur with the Unite for Sight^35^ findings that CHW programmes are hindered by incorrect design and implementation of training programmes, which creates barriers for CHWs in completion of their duties. Community health workers felt that they lacked knowledge and skill in responding to childhood disorders, for which they needed further training to be able to deliver a better service.^36^ Community health worker supervisors noted that there were varying levels of training amongst the CHWs and that only the younger CHWs made use of technology to facilitate knowledge acquisition whilst the senior CHWs relied on information received only from relevant stakeholders. Several authors suggest that any curriculum needs to cover all the roles and tasks allocated to a specific group, with service providers needing to develop a procedural handbook to facilitate understanding of basic first aid, support screening in school, the work in Phila Mntwana centres and in the provision of psychosocial counselling.^2,32,34^ This study’s findings further suggest that the CHWs could have procedures in place to allow them to recognise when assistive devices are required, for carry-over of rehabilitation and early identification of children at risk of developmental delays and disabilities. Additionally, the fact that CHWs receive no specific training to assess, handle and refer at-risk children or children with a disability indicates that the needs of the community are not being addressed. There is a critical need for training in identifying and dealing with childhood disorders and disabilities, including basic information regarding identification of different childhood disorders and basic handling skills so that CHWs can educate parents and caregivers in their home environments. Still, CHWs need training on the role of the various health care professions and their role, as well as the resources available in the area (schools, support groups, etc.) to ensure that they make appropriate referrals to the relevant stakeholders. Furthermore, CHWs would benefit from first aid training, as they are often the first person on the scene but do not have the knowledge or skills to deal with emergencies as first responders. Community health workers and key informants also indicated that future training plans must include sign language, physiotherapy exercises, epilepsy, mental health and how to assist a blind person. This study suggests that CHWs need to receive information sessions as part of work-integrated learning from the multidisciplinary health team to implement the WBOT policy^3^ vision of CHWs being able to deliver health promotion, disease prevention, therapeutic and palliative care services.

## Conclusion

In this study, the challenges of the roles of CHWs were acknowledged, especially in terms of training needs in order to provide effective services to children with disorders and disabilities. A CHW is an invaluable asset that assists in linking sectors that work together in the health system and is integral to the extension of health care. Although the eThekwini CHW programme has the potential to serve as a cost-effective means of tracking children with childhood disorders and disabilities, this study indicates that there is a critical need for training in this area for the programme to be effective. It is essential that CHWs be more suitably trained, as they have better access to children in the community to ensure that all children who need access to health services receive care rather than being lost in the system. This would assist in ensuring adherence to the DoH national service delivery mandate of improving child longevity and health. Whilst this study was limited to only three communities in one district of a province in the country, the incongruences in supervision and training, lack of role definition and scope of practice has highlighted critical areas for intervention. This remains a challenge not only for the management of childhood disorders but in the holistic role of this cadre of health worker, who inevitably is an extension of the health care team.
